# Hyperspectral Image Denoising via Quasi-Recursive Spectral Attention and Cross-Layer Feature Fusion

**DOI:** 10.3390/s25226955

**Published:** 2025-11-14

**Authors:** Yanhua Xiao, Huayan Zhou, Wenfeng Li, Long Yang, Ke Wang

**Affiliations:** 1 School of Information Engineering, Chenzhou Vocation Technical College, Chenzhou 424500, China; 2 State Key Laboratory of Medical Neurobiology, MOE Frontiers Center for Brain Science, Fudan University, Shanghai 200433, China; 3 College of Computer and Software, Chengdu Jincheng College, Chengdu 611731, China; wangke@cdjcc.edu.cn

**Keywords:** hyperspectral image denoising, spatial–spectral modeling, quasi-recurrent attention, multi-head spectral attention, cross-layer skip connection

## Abstract

Hyperspectral images (HSIs) contain rich spatial–spectral information but are highly susceptible to various types of noise during the imaging process, which significantly degrades image quality and undermines the reliability of subsequent applications. To address this issue, we propose a novel end-to-end denoising framework, termed Quasi-Recursive Spectral Attention Network (QRSAN), which aims to design a feature extraction module that leverages the intrinsic characteristics of hyperspectral noise while preserving high-quality spatial and spectral information. Specifically, QRSAN introduces a Quasi-Recursive Attention Unit (QRAU) to jointly model inherent spatial–spectral dependencies, where 2D convolutions are employed for spatial feature extraction and frequency pooling is utilized for spectral representation. In addition, we develop a multi-head spectral attention mechanism to effectively capture inter-band correlations and suppress spectrally dependent noise. To further preserve fine-grained spatial structures and spectral fidelity, we design an adaptive cross-layer skip connection strategy that integrates channel-wise concatenation and transition blocks, enabling efficient feature propagation and fusion within an asymmetric encoder–decoder architecture. Extensive experiments on both synthetic and real HSI datasets demonstrate that QRSAN consistently outperforms existing methods in terms of visual quality and objective evaluation metrics, achieving superior denoising performance and generalization ability while maintaining high spatial–spectral fidelity.

## 1. Introduction

Hyperspectral images (HSIs), which provide both fine-grained spatial details and continuous spectral information, have been widely applied in various fields such as remote sensing [1], medicine [2], agriculture [3], and food inspection [4]. By simultaneously recording spatial and spectral responses across hundreds of contiguous bands, HSIs enable accurate material identification and discrimination, which makes them uniquely advantageous for vision tasks involving detailed spatial–spectral feature extraction. Consequently, HSIs have been extensively used in image classification [5,6,7], object detection [8,9], object tracking [10,11], change detection [12,13], and anomaly detection [14,15], demonstrating their irreplaceable value in both academic research and real-world applications. However, during the acquisition process, HSIs are inevitably contaminated by various degradation factors, including insufficient exposure, platform jitter, atmospheric disturbance, photon counting errors, stray light [16], and environmental noise. These physical limitations often introduce different noise patterns into HSIs, such as Gaussian noise, impulse noise, stripe noise, and dead-line noise [17,18,19]. Notably, degradations caused by stray light—such as ghost reflections or scattering that deteriorate the performance of optical instruments [16]—often affect multiple spectral bands simultaneously, thereby leading to complex mixed noise distributions. Conventional hyperspectral imaging systems are generally based on pure amplitude imaging, where only the intensity of the reflected or emitted light is recorded. In contrast, complex-valued hyperspectral imaging techniques [20] extend this framework by capturing both amplitude and phase information, enabling more comprehensive spectral–spatial characterization and improved material discrimination. Despite their theoretical advantages, complex-valued HSIs remain highly susceptible to severe noise issues, including phase instability, quantization artifacts, and optical interference, which can significantly distort spectral reconstruction. Therefore, robust denoising remains a critical prerequisite for ensuring reliable spectral analysis and high-quality interpretation in both amplitude-only and complex-valued HSI modalities.

Over the past decade, extensive research has been devoted to HSI denoising, and existing methods can generally be grouped into two categories: model-based approaches and learning-based approaches. Model-based approaches typically formulate the denoising task as an inverse problem, regularized by suitable prior constraints to make the ill-posed problem tractable. For example, representative priors include  [21,22,23], which exploits the sparsity of images in a specific transform or dictionary domain to separate noise from essential features for effective reconstruction; nonlocal similarity [24,25,26], which leverages the repetition of similar patches across spatial or spectral dimensions and aggregates them to enhance denoising; total variation (TV) regularization [27,28,29], which constrains the gradient magnitude to preserve edges while suppressing noise in smooth regions; and low-rank properties [30,31,32], which model hyperspectral data as low-rank matrices or tensors by exploiting strong spectral correlations, enabling noise removal while maintaining structural information. Representative algorithms such as BM4D [24], VBM4D [33], CDBM3D [34], CCF [35], tensor dictionary learning (TDL) [36], and low-rank tensor recovery (LLRT) [31] have shown effectiveness by exploiting the global spectral correlation (GSC) and spatial nonlocal self-similarity (NSS) of HSIs. These approaches are physically interpretable and well-founded in theory, but they also come with critical limitations: they heavily depend on handcrafted assumptions, usually require iterative solvers, and often suffer from high computational complexity and weak generalization ability when applied to diverse real-world noise.

With the success of deep learning, particularly convolutional neural networks (CNNs), learning-based approaches have attracted significant attention in recent years [37,38]. Unlike model-driven methods that rely on explicit priors, CNN-based frameworks directly learn implicit feature priors from paired noisy–clean data [39,40,41]. Such end-to-end models offer higher flexibility, generalization, and efficiency, thereby alleviating the dependence on physical degradation models. However, CNNs are inherently limited by their local connectivity and fixed convolutional kernels. Their finite receptive field and weight-sharing property make them less suitable for modeling sequential spectral data, often leading to insufficient robustness against complex noise patterns [42]. As a result, CNN-based methods may struggle to preserve subtle spatial–spectral structures in large-scale HSIs, especially under strong or mixed noise conditions.

More recently, attention mechanisms and Transformer architectures have been introduced into HSI denoising. Compared with CNNs, self-attention is capable of effectively modeling long-range dependencies and enhancing global feature representations, which has led to superior performance in various computer vision tasks. Moreover, the weaker inductive bias of Transformers allows them to better exploit large-scale data, thereby overcoming the receptive field and weight-sharing constraints of CNNs [43,44,45]. Representative architectures such as Swin Transformer and U-shaped Transformer networks have demonstrated their effectiveness in image restoration and reconstruction and have gradually been extended to HSI denoising. Nevertheless, Transformers still face notable challenges: their ability to capture local features is limited, making it difficult to fully exploit spatial nonlocal similarity; the fully connected attention mechanism is prone to noise interference during feature aggregation; and their quadratic computational complexity with respect to input resolution greatly restricts their applicability to high-resolution HSIs.

In summary, the development of HSI denoising has progressed from traditional model-based methods with handcrafted priors to CNN-based frameworks that automatically learn discriminative features and further to Transformer-based models capable of global dependency modeling. Despite these advances, achieving a balance between denoising accuracy, computational efficiency, and robustness to diverse noise types remains a fundamental challenge in this field.

To address these issues, we propose a novel end-to-end denoising network, termed the Quasi-Recursive Spectral Attention Network (QRSAN). The key idea is to explicitly leverage spatial–spectral correlations while maintaining computational efficiency. Within QRSAN, we introduce the Quasi-Recursive Attention Unit (QRAU), which employs 2D convolutions to extract local spatial features and integrates frequency pooling along the spectral dimension to model inter-band redundancy. In particular, considering the strong noise dependency across adjacent bands, we design a multi-head spectral attention mechanism to strengthen inter-band feature correlation and suppress structured noise. Furthermore, to preserve low-level structural details that are crucial for reconstruction, we propose a cross-layer skip connection strategy with channel concatenation and a transition block, enabling effective multi-level feature propagation and improving both spatial fidelity and spectral consistency. Extensive experiments conducted on multiple benchmark datasets demonstrate that QRSAN achieves superior performance compared with state-of-the-art methods, validating its effectiveness and robustness in practical HSI denoising scenarios.The main contributions of this work are summarized as follows:

1. A novel QRSAN architecture is proposed, consisting of multiple QRAUs that effectively explore intrinsic spatial–spectral features of HSIs and precisely capture noise dependencies across adjacent bands.
2. A channel concatenation strategy with dedicated transition blocks is introduced to facilitate feature propagation, enabling multi-level feature fusion within an asymmetric encoder–decoder architecture, thus preserving structural consistency and enhancing spatial–spectral fidelity.
3. Comprehensive experiments on diverse synthetic and real HSI denoising tasks demonstrate that QRSAN consistently outperforms existing methods in terms of both denoising performance and generalization ability, validating its effectiveness and superiority.

The remainder of this paper is organized as follows. Section 2 presents the proposed method. Section 3 reports the experimental results, and Section 4 concludes the paper.

## 2. Methods

### 2.1. Notations

Let the clean hyperspectral image be $X\in R^{H\times W\times B}$, where *H* and *W* are the spatial dimensions and *B* is the number of spectral bands, with each pixel represented by a *B*-dimensional spectral vector. In practice, HSIs are inevitably degraded during acquisition due to sensor limitations, environmental interference, or transmission errors, which can be modeled as an additive noise *E*, yielding the observed image Y=X+E. The noise may vary across spectral bands and take diverse forms, making denoising challenging. The goal of HSI denoising is thus to recover the clean image *X* from *Y* while preserving both spatial structures and spectral fidelity.

### 2.2. Training Loss Function

The proposed QRSAN aims to learn a mapping function from the degraded image to the clean image, thereby achieving hyperspectral image denoising and reconstruction. The training objective is to minimize the $L_{2}$ distance between the predicted image $\hat{X}$ and the ground truth *X*, with the loss function defined as follows:

(1) $$L=\frac{1}{N}\sum_{i=1}^{N}{∥{\hat{X}}_{i}-X_{i}∥}_{F}^{2},$$

Here, *N* denotes the batch size within each iteration.

### 2.3. Overall Architecture

**Figure 1:**
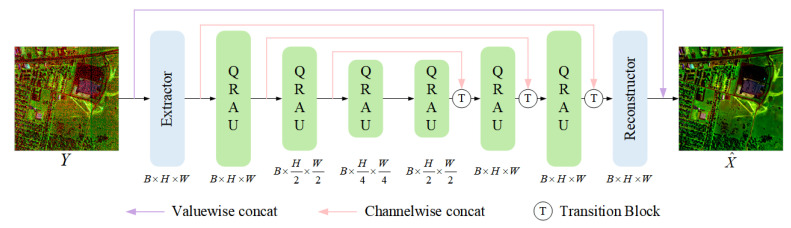
Architectural Details of QRSAN.

The overall architecture of the proposed QRSAN is illustrated in Figure 1. To fully exploit the feature modeling capability of the Quasi-Recursive Attention Unit (QRAU) and to achieve high-fidelity HSI reconstruction, the network adopts an encoder–decoder framework, which has proven effective in balancing representation learning and detail recovery. The backbone of QRSAN is constructed with three pairs of symmetric QRAU layers, forming a hierarchical structure that progressively extracts abstract representations while preserving fine spatial–spectral information. On top of this backbone, several specifically designed modules are integrated to further enhance denoising performance and maintain spatial–spectral consistency.

First, let the input HSI feature map be $X\in R^{C_{in}\times B\times H\times W}$ and the output feature map after 3D convolution be $X^{\prime }\in R^{C_{out}\times B\times H^{\prime }\times W^{\prime }}$. The convolution is applied as

(2) $$X^{\prime }\left(:,b,:,:\right)=\sum_{c=1}^{C_{in}}X\left(c,b,:,:\right)*K_{c},\;b=1,2,\ldots ,B,$$

where $K_{c}\in R^{k_{H}\times k_{W}}$ is the 2D spatial kernel, ∗ denotes 2D convolution, and the stride along the spectral dimension is fixed to 1 with kernel size $k_{B}=1$, ensuring independent processing of each spectral band while preserving spectral continuity. This design ensures that spectral continuity is preserved without introducing band mixing, while also eliminating restrictions on the number of spectral channels. As a result, the model is highly flexible and can be directly applied to hyperspectral datasets with arbitrary numbers of bands, from tens to hundreds, without the need for reconfiguration or retraining.

Second, as the network depth increases, the receptive field of feature maps expands, allowing the model to capture long-range dependencies. However, this comes at the cost of gradually losing fine structural details, especially in high-frequency regions. Since reliable denoising requires compensating for the information loss introduced by downsampling and deeper transformations, skip connections play a crucial role in feature preservation. Instead of using standard symmetric skip connections, we design an asymmetric skip connection strategy tailored to hyperspectral noise characteristics. This strategy consists of channelwise concatenation and transition blocks, which fuse multi-scale features from corresponding encoder and decoder stages. In doing so, the proposed design not only helps retain spatial edges and spectral signatures but also alleviates optimization difficulties such as gradient vanishing or explosion, thus stabilizing network training.

Furthermore, the asymmetric skip connections enhance cross-layer feature interaction, allowing low-level structural cues and high-level semantic features to be jointly exploited during reconstruction. This ensures that both local spatial textures and global spectral correlations are preserved, which is particularly important for denoising tasks where over-smoothing or spectral distortion can easily occur.

In summary, beyond the efficient noise modeling provided by QRAUs, the proposed asymmetric QRSAN architecture integrates multi-scale contextual information with carefully designed skip connections, thereby preserving high-resolution structures and richer details during reconstruction. This collaborative design enables QRSAN to achieve superior denoising performance, demonstrating its robustness and generalization ability across diverse HSI noise scenarios.

### 2.4. Quasi-Recurrent Attention Unit

**Figure 2:**
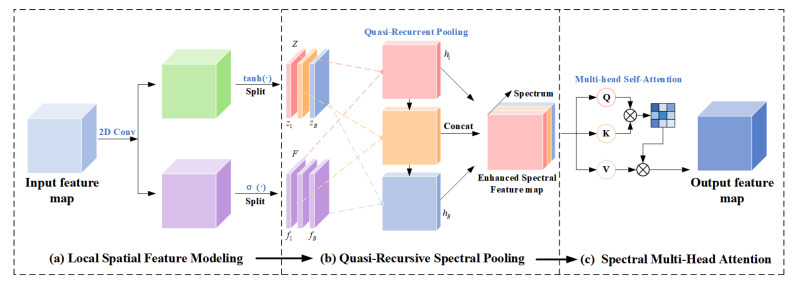
Design details of the QRAU. It mainly consists of three components: (**a**) Local Spatial Feature Modeling, (**b**) Quasi-Recursive Spectral Pooling, and (**c**) Spectral Multi-Head Attention.

Effective feature extraction is essential for reconstructing clean hyperspectral images (HSIs). Since noise distributions vary across spectral bands and may involve multiple types or intensities, it is necessary to capture not only local spatial features but also long-range spectral dependencies during modeling. To this end, we design the Quasi-Recurrent Attention Unit (QRAU), which integrates lightweight convolutional operations with recursive spectral modeling and attention mechanisms. This design allows for efficient joint spatial–spectral feature extraction while maintaining low computational overhead. The structure of the QRAU is illustrated in Figure 2.

#### 2.4.1. Local Spatial Feature Modeling

HSIs exhibit strong nonlocal similarity in the spatial domain, and multi-scale contextual information plays a critical role in both denoising and reconstruction. A straightforward solution is to employ multi-scale convolutional kernels to capture different receptive fields. However, this approach significantly increases the number of parameters and computational complexity. To strike a balance between performance and efficiency, we instead introduce multi-resolution inputs through scaling operations in the data augmentation stage, followed by a fixed-scale convolutional backbone for feature extraction. This strategy improves the diversity of training samples while avoiding redundant convolutional operations.

As shown in Figure 2a, we apply independent 2D convolutional kernels in parallel to each spectral band, thereby enabling effective spatial feature extraction without mixing band information. Formally, given an input feature map $X\in R^{C_{in}\times B\times H\times W}$(where, in the first layer, *X* corresponds to the original HSI patch with $C_{in}=1$), two parallel convolutional branches are constructed to generate a candidate tensor $Z\in R^{C_{out}\times B\times H\times W}$ and a forget gate $F\in R^{C_{out}\times B\times H\times W}$:

(3) $$Z=\tanh(W_{z}*X),$$

(4) $$F=\sigma (W_{f}*X),$$

where $W_{z}$ and $W_{f}$ are convolutional filter banks, each of size 1×3×3, and ∗ denotes 2D convolution. The tanh activation ensures nonlinearity in candidate features, while the sigmoid gate regulates information flow.

#### 2.4.2. Quasi-Recursive Spectral Pooling

In addition to spatial correlation, HSIs also exhibit strong spectral redundancy, which has often been modeled using low-rank priors in traditional methods. However, low-rank modeling alone tends to oversimplify spectral variations and may lose fine-grained details. To better exploit spectral correlation, we propose a quasi-recursive pooling mechanism along the spectral dimension.

As shown in Figure 2b, the candidate tensor *Z* and forget gate *F* are decomposed into band-wise sequences $z_{i}$ and $f_{i}$, which are updated sequentially:

(5) $$h_{i}=f_{i}⊙h_{i-1}+\left(1-f_{i}\right)⊙z_{i},\forall i\in \left[1,B\right],$$

where ⊙ denotes elementwise multiplication and $h_{i}$ represents the hidden state of the *i*-th band (initialized to zero). In this formulation, the forget gate $f_{i}\in \left[0,1\right]$ adaptively balances the current band representation $z_{i}$ and the historical state $h_{i-1}$.

This recursive update ensures that information flows progressively along the spectral dimension, while the gating mechanism prevents error accumulation. Compared to strict recurrent formulations (e.g., RNN or LSTM), the proposed quasi-recursive pooling avoids full sequential dependency, thereby mitigating gradient vanishing and reducing computational cost while maintaining inter-band continuity [46]. Unlike 3D CNNs that model spatial–spectral cubes using fixed convolutional kernels, the quasi-recursive mechanism adaptively adjusts the contribution of each band through dynamic gating, leading to more flexible and context-aware spectral modeling [41]. After processing all bands, the hidden states $h_{i}$ are concatenated to form the enhanced spectral feature map *F*, which serves as the input to the subsequent spectral attention module. This design ensures that the quasi-recursive pooling stage provides a compact and context-aware spectral representation, which is then refined by the attention mechanism for global spectral dependency modeling.

#### 2.4.3. Spectral Multi-Head Attention

While quasi-recursive pooling captures local and sequential dependencies across adjacent bands, it is insufficient for modeling long-range spectral dependencies, especially when noise patterns exhibit cross-band correlations. To address this, we incorporate a spectral multi-head attention mechanism into QRAU.

In Figure 2c, taking the enhanced spectral feature map *F* from the quasi-recursive pooling stage as input, each spectral band is projected into three learnable representations: queries (*Q*), keys (*K*), and values (*V*). These are used to compute attention weights across all bands in parallel to capture global inter-band relationships:

(6) $$\hat{F}=W_{p}\cdot \mathrm{Attention}\left(\hat{Q},\hat{K},\hat{V}\right)+F,$$

(7) $$\mathrm{Attention}\left(\hat{Q},\hat{K},\hat{V}\right)=\hat{V}\cdot \mathrm{Softmax}\left(\frac{\hat{Q}\cdot \hat{K}}{\alpha }\right),$$

Here, α is a learnable scaling factor that stabilizes the magnitude of the dot product; $W_{p}$ denotes a linear projection matrix used to aggregate the attended features; Softmax normalizes across all spectral band indices i=1,2,…,B to ensure the attention weights sum to 1; and Attention represents the resulting weighted output features for each band.

Through this design, the attention mechanism adaptively assigns correlation weights to different bands, thereby reinforcing informative bands while suppressing noisy ones. Moreover, the multi-head formulation enhances nonlinear modeling capacity and ensures that different subspaces of spectral dependencies can be captured simultaneously.

In summary, the proposed QRAU integrates local spatial convolutions, quasi-recursive spectral pooling, and multi-head spectral attention in a lightweight yet powerful framework. The overall data flow of QRAU follows a sequential structure, where local spatial features are first extracted and modulated by band-wise gating, followed by quasi-recursive pooling for local spectral correlation modeling, and finally by multi-head attention for global spectral refinement. This hierarchical design clarifies the interaction between the quasi-recursive and attention components, ensuring smooth spectral information propagation. This combination allows QRAU to adaptively retain clean band information, suppress noise in corrupted bands, and model both local and global spectral dependencies. As a result, QRAU serves as an effective building block for QRSAN, enabling robust spatial–spectral feature learning and high-fidelity HSI reconstruction.

### 2.5. Transition Block

**Figure 3:**
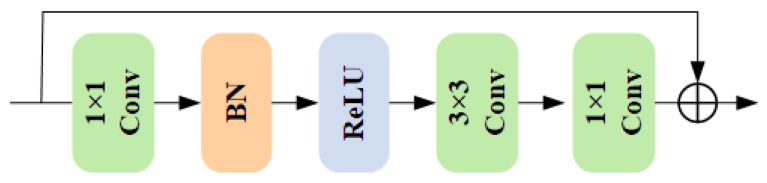
Design Details of the Transition Block.

The internal structure of the Transition Block, as illustrated in Figure 3, consists of two 1×1 convolutional layers and a BN–ReLU–Conv sequence, with residual connections added before and after the 1×1 convolutions. Formally, the computation can be expressed as

(8) $$Y=X+Conv_{1\times 1}^{\left(2\right)}*\left(Conv_{3\times 3}*\delta \left(\mathrm{BN}\left(Conv_{1\times 1}^{\left(1\right)}*X\right)\right)\right),$$

where *X* denotes the input feature map and * indicates the convolution operation. Here, Conv represents a convolutional layer used to extract and transform spatial and spectral features; $Conv_{1\times 1}^{\left(1\right)}$ compresses the channel dimension to reduce computational cost and generate compact representations, while $Conv_{1\times 1}^{\left(2\right)}$ restores and remaps the channels after feature fusion. The 3×3 convolution enhances the interaction of low-level spatial details and spectral information. BN (Batch Normalization) standardizes the layer output to improve training stability and convergence, and δ denotes the ReLU (Rectified Linear Unit) activation function, defined as δ(z)=max(0,z), which introduces nonlinearity to enhance model expressiveness. Residual addition ensures stable gradient propagation and preserves consistency between input and output.

The proposed algorithm in this paper is shown in Algorithm 1.
**Algorithm 1:** HSI Denoising with the QRSAN Algorithm.
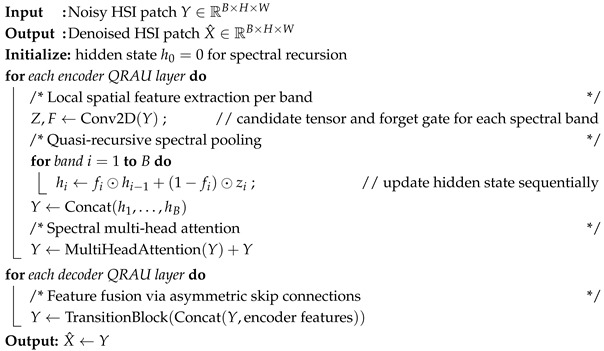


## 3. Experiments

To evaluate the effectiveness of the proposed method, we conducted extensive experiments on both synthetic and real-world datasets. Seven representative denoising algorithms were selected for comparison, including three model-based approaches—BM4D [24], NGMeet [26], and LRTFL0 [47]—as well as four deep learning-based methods—QRNN3D [41], T3SC [48], MAC-Net [49], and SST [45]. For fairness, all learning-based baselines were retrained and tested under the same settings. The proposed model was implemented in PyTorch 2.6.0+cu126 and optimized with Adam using a learning rate of $1\times 10^{-4}$. Training was performed on a single NVIDIA GeForce RTX 3090 GPU (NVIDIA, Santa Clara, CA, USA).

### 3.1. Simulated HSI Experiments

In this study, we conducted simulation experiments on the ICVL dataset, where the original HSIs were regarded as clean references. The ICVL dataset consists of 201 hyperspectral images captured by a Specim PS Kappa DX4 hyperspectral camera (Specim, Oulu, Finland), with a spatial resolution of pixels and 31 spectral bands covering the wavelength range of 400–700 nm.

To simulate diverse noise conditions, two types of degradations were introduced: Gaussian noise and mixed noise.

For complex noise, four settings were considered, including three fixed noise levels (σ=30, σ=50, and σ=70) and one blind scenario (σ∈[30,70]), enabling a comprehensive evaluation of robustness under varying noise intensities.

For complex noise, five representative scenarios were considered:

Case 1 (Non-i.i.d. Gaussian Noise): Zero-mean Gaussian noise with band-dependent intensities randomly sampled from [10%, 70%] is added to each spectral band.

Case 2 (Gaussian + Stripe Noise): Building on Case 1, stripe noise with intensity in [5%, 15%] is added to a randomly selected one-third of the bands.

Case 3 (Gaussian + Deadline Noise): Based on Gaussian noise, deadline noise with intensity in [5%, 15%] is introduced to one-third of the bands randomly.

Case 4 (Gaussian + Impulse Noise): In addition to Gaussian noise, impulse noise with intensity randomly sampled from [10%, 70%] is injected into one-third of the bands.

Case 5 (Mixture Noise): Gaussian noise is added to all bands, while one-third of the bands are randomly corrupted with a combination of the four aforementioned noise types, simulating a more complex and realistic noise environment.

Furthermore, we employed three commonly used metrics—PSNR, SSIM, and SAM—to evaluate the performance of each model in the synthetic experiments and also reported the computational time for each method. The first two metrics assess spatial similarity, while SAM quantifies spectral consistency. Given a reference image and a reconstructed image, PSNR is computed as

(9) $$\mathrm{PSNR}=10\log_{10}\left(\frac{{\left(2^{n}-1\right)}^{2}}{\mathrm{MSE}}\right),$$

(10) $$\mathrm{MSE}=\frac{1}{HW}\sum_{i=1}^{H}\sum_{j=1}^{W}{\left[I\left(i,j\right)-\hat{I}\left(i,j\right)\right]}^{2},$$

Here, *H* and *W* denote the height and width of the image, respectively, and *n* represents the number of possible pixel values, which is typically 8.

The core formulas of SSIM and SAM are given as follows:

(11) $$\mathrm{SSIM}=\frac{\left(2\mu_{I}\mu_{\hat{I}}+c_{1}\right)\left(2\sigma_{I\hat{I}}+c_{2}\right)}{\left(\mu_{I}^{2}+\mu_{\hat{I}}^{2}+c_{1}\right)\left(\sigma_{I}^{2}+\sigma_{\hat{I}}^{2}+c_{2}\right)},$$

(12) $$\mathrm{SAM}=\arccos\left(\frac{\langle I,\hat{I}\rangle }{∥I∥∥\hat{I}∥}\right),$$

Here, $\mu_{I}$ and $\mu_{\hat{I}}$ denote the mean values of the reference image *I* and the reconstructed image $\hat{I}$, respectively; $\sigma_{I}^{2}$ and $\sigma_{\hat{I}}^{2}$ represent their variances, and $\sigma_{I\hat{I}}$ is the covariance between *I* and $\hat{I}$; $c_{1}$ and $c_{2}$ are small constants introduced to avoid division by zero; $\langle I,\hat{I}\rangle$ denotes the inner product between the spectral vectors of the reference and reconstructed images; and ∥·∥ represents the Euclidean norm. Higher PSNR and SSIM values, along with lower SAM values, indicate better model performance. Since HSIs contain hundreds of spectral bands, all metrics are computed for each band and the final results are obtained by averaging across bands.

#### 3.1.1. Denoising Under Gaussian Noise Conditions

The quantitative comparison of several HSI denoising methods is presented in Table 1. The results demonstrate that QRSAN consistently achieves superior performance across all noise levels as well as in the blind scenario. QRSAN maintains an advantage in all three core metrics—PSNR, SSIM, and SAM—highlighting its effectiveness in restoring image quality while preserving spectral fidelity. Other deep learning-based methods, such as SST and MAC-Net, also perform well, further confirming the significant potential of data-driven approaches in hyperspectral image denoising.

**Figure 4:**
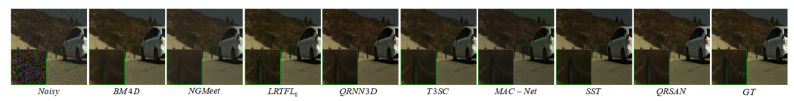
Visual comparison of different methods on the ICVL negev_0823-1003 data at bands (28, 18, and 8) with Gaussian noise level σ=50.

To visually illustrate the effectiveness of QRSAN, Figure 4 presents denoising results under a noise level of, with key regions magnified for detailed comparison. Among traditional model-based methods, NGMeet effectively suppresses noise by leveraging non-local self-similarity priors but tends to over-smooth complex textured regions. LRTF$L_{0}$ preserves some texture details yet still leaves residual noise. In contrast, deep learning-based approaches, benefiting from strong data-driven capabilities, outperform traditional methods across different noise levels. Notably, QRSAN achieves a superior balance between noise suppression and detail preservation, effectively enhancing both the spectral fidelity and the overall visual quality of the reconstructed images.

#### 3.1.2. Denoising Under Complex Noise Conditions

Table 2 presents the quantitative evaluation of various methods under five representative noise types: Non-i.i.d. Gaussian, Stripe, Deadline, Impulse, and Mixture noise.

The results indicate that QRSAN achieves the highest or near-highest PSNR and SSIM values across all scenarios, while attaining the best performance in SAM, demonstrating superior image reconstruction quality, spectral fidelity, and robust stability. In comparison, SST excels in structural similarity and detail preservation, particularly showing advantages in SSIM and SAM metrics. QRNN3D, T3SC, and MAC-Net exhibit relatively balanced performance under diverse noise conditions. Traditional model-based methods such as BM4D, NGMeet, and LRTF$L_{0}$ perform moderately, with noticeable limitations when handling complex noise. Overall, deep learning-based approaches outperform conventional methods in hyperspectral image denoising tasks, with QRSAN standing out due to its superior fidelity and stronger noise suppression capability.

**Figure 5:**
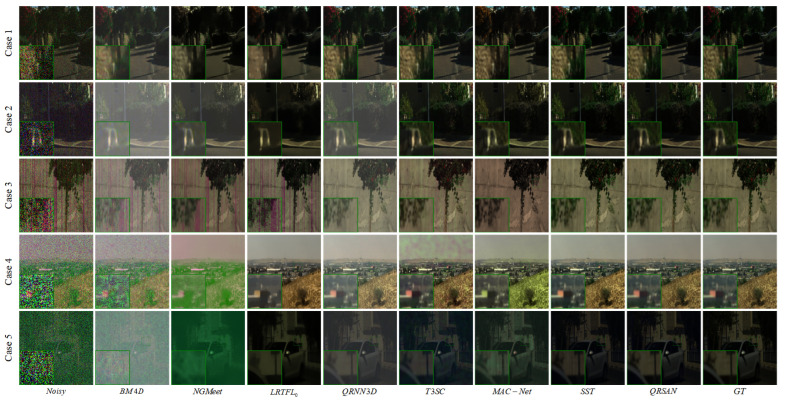
Visual comparison of different methods on the ICVL dataset under Case 1–Case 5.

Figure 5 illustrates representative HSI samples under different noise scenarios along with the denoising results of each method. For more intuitive comparison, key regions are magnified to highlight differences in structure preservation and noise suppression among the methods.

### 3.2. Real HSI Experiments

#### 3.2.1. Urban

To further evaluate the denoising performance of the QRSAN algorithm, additional experiments were conducted on the Urban dataset, acquired with the HYDICE sensor (The U.S. Army Research Laboratory, Adelphi, MD, USA). This dataset contains 210 spectral bands with a spatial resolution of 307 × 307 pixels and covers the 400–2500 nm spectral range. Several bands are affected by atmospheric interference and exhibit mixed noise, including Gaussian, stripe, and dead-line noise.

**Figure 6:**
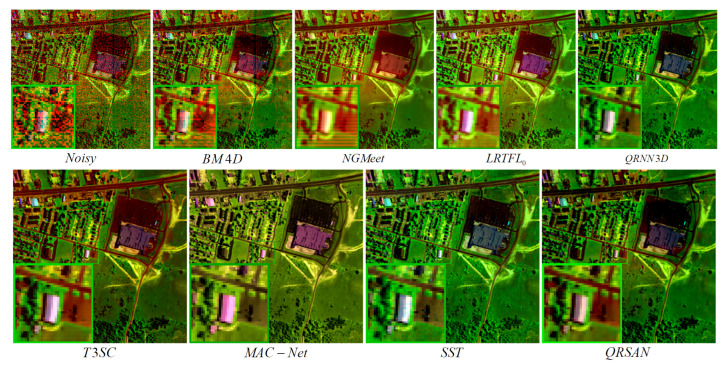
Visual comparison of real denoising results on the Urban dataset at bands (208, 101, and 1).

**Figure 7:**
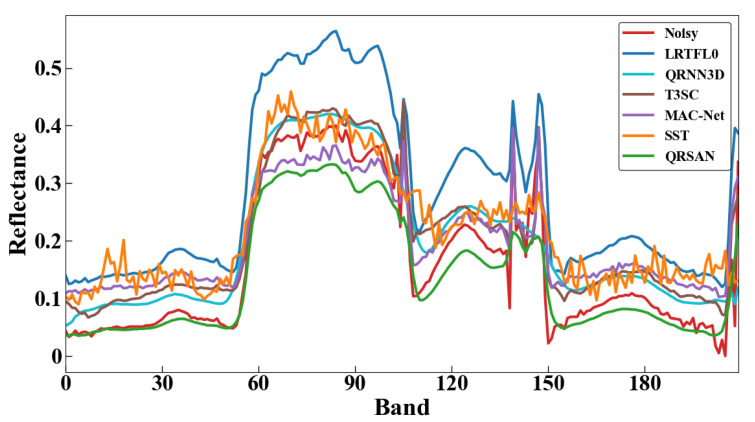
The reflectance of pixel (80, 120) in the Urban HSI.

Figure 6 shows the real-world denoising results of QRSAN compared with seven benchmark methods on the Urban dataset. QRSAN effectively suppresses mixed noise while retaining fine spatial details, demonstrating superior overall performance. Among the model-based methods, NGMeet utilizes non-local self-similarity priors and reduces noise but tends to over-smooth complex textures, whereas LRTF$L_{0}$ achieves relatively good results in mixed noise removal. Deep learning-based methods, including QRNN3D, T3SC, and MAC-Net, generally remove noise effectively; however, QRNN3D loses some texture details, and T3SC and MAC-Net exhibit certain spectral distortions. SST struggles to fully remove stripe noise, indicating limited adaptability to challenging imaging conditions. Figure 7 presents the corresponding spectral reflectance curves. Overall, QRSAN achieves a better balance between noise suppression, detail preservation, and spectral fidelity than the compared methods.

#### 3.2.2. Realistic Dataset

The Realistic dataset [50] comprises 59 paired noisy and clean HSIs, each with a spatial resolution of 696 × 520 pixels and 34 spectral bands covering 400–700 nm. It serves as a standard benchmark for evaluating real-world hyperspectral denoising performance.

**Figure 8:**
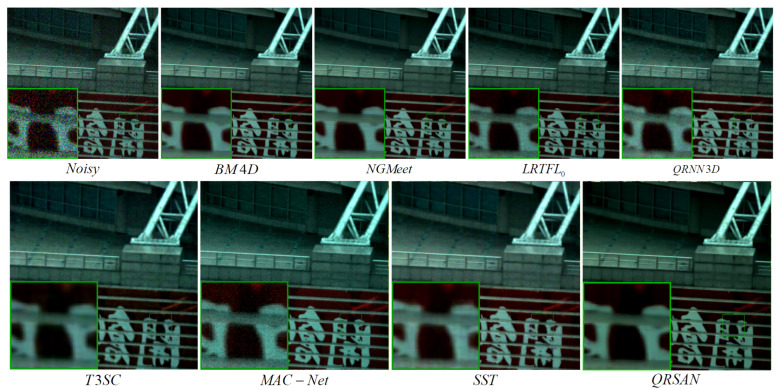
Real denoising results on bands (30, 18, and 15) of Scene 2 in the Realistic dataset.

Figure 8 presents the denoising results of various methods on the Realistic dataset under real-world conditions. As reported in Table 3, QRSAN achieves the highest PSNR and SSIM values along with the lowest SAM, demonstrating superior performance in image quality restoration, structural preservation, and spectral fidelity. SST follows closely, effectively balancing noise reduction and detail retention, indicating its capability in preserving spatial textures and spectral smoothness. Methods such as MAC-Net, T3SC, and QRNN3D show relatively stable performance across different noise types and levels, maintaining a reasonable trade-off between detail preservation and noise suppression. Model-based approaches, including BM4D, NGMeet, and LRTF$L_{0}$, offer certain spectral consistency advantages but are limited in modeling complex noise, resulting in less effective detail recovery and overall image enhancement. To verify the reliability of the performance improvement, a paired *t*-test between QRSAN and SST was conducted on the Realistic dataset, and the results confirm that the improvements in PSNR, SSIM, and SAM are statistically significant (p<0.05).

### 3.3. Ablation Study

In this section, we evaluate the effectiveness of each component of QRSAN on the ICVL dataset and explore the optimal trade-off between denoising performance and computational cost. PSNR, SSIM, and the total number of network parameters are used as the evaluation metrics.

#### 3.3.1. Effectiveness of QRAU Components

To comprehensively assess the contribution of the QRAU module within QRSAN, ablation experiments were conducted on the proposed Quasi-Recursive Attention Unit and its variants, RES2D, QRU2D, QRU3D, and an LSTM-based recurrent unit (denoted as LSTM), as summarized in Table 4. RES2D removes both the gated quasi-recursive pooling and spectral attention, QRU2D combines 2-D convolutions with quasi-recursive pooling, and QRU3D extends QRU2D using 3-D convolutions. The LSTM variant replaces QRAU with a fully recurrent spectral model under the same framework and training settings.

As shown in Table 4, RES2D exhibits substantially lower performance, highlighting the importance of spectral modeling. QRU3D improves over QRU2D due to 3-D convolutions but lacks spectral attention. The LSTM-based recurrent unit achieves slightly better performance than QRU3D, benefiting from its explicit modeling of long-range spectral dependencies. However, it incurs more parameters and higher computational cost and lacks flexibility in adapting to arbitrary numbers of spectral bands. In contrast, the proposed QRAU integrates lightweight 2-D convolutions with multi-head spectral attention, capturing both spatial and spectral dependencies effectively. This quasi-recursive design provides greater flexibility and robustness, enabling adaptive denoising across diverse noise types and HSIs with varying spectral dimensions, while remaining computationally efficient.

#### 3.3.2. Skip Connections

Table 5 presents a comparison of different skip connection strategies. Specifically, N-net employs no skip connections, V-net uses progressive additive skip connections, and C-net incorporates channel-wise concatenation combined with transition blocks for feature propagation. The results indicate that N-net performs the worst, demonstrating that the absence of skip connections leads to the loss of high-level information. Both V-net and C-net outperform N-net significantly with comparable computational costs.

Notably, V-net is more lightweight, whereas C-net achieves superior denoising performance, suggesting that the channel-wise concatenation and transition block design provides an effective alternative to conventional skip connections. Considering the trade-off between performance and computational efficiency, QRSAN adopts C-net within the encoder–decoder framework while employing V-net to bridge shallow feature extraction and final reconstruction. The final configuration achieves state-of-the-art denoising results.

### 3.4. Limitations and Potential Impact

While QRSAN demonstrates strong performance in hyperspectral image denoising across synthetic and real-world datasets, several limitations exist. Its robustness under extremely high noise levels or rare, unseen noise types remains uncertain, and performance may degrade with severely corrupted bands. The generalization to hyperspectral images from sensors with different spectral ranges or imaging conditions is not fully validated, and domain shifts may affect spectral fidelity. Like other deep learning methods, QRSAN relies on sufficient labeled data, and computational costs may limit real-time processing of large datasets. Despite these challenges, the quasi-recursive pooling and spectral attention mechanisms provide a flexible framework for spectral–spatial modeling, with potential extensions to tasks such as anomaly detection, unmixing, and super-resolution. Future work may explore domain adaptation, self-supervised learning, or noise-aware strategies to improve robustness and cross-sensor generalization.

## 4. Conclusions

This work introduces the Quasi-Recursive Spectral Attention Network (QRSAN) for hyperspectral image (HSI) denoising, designed to exploit HSI noise characteristics for efficient spatial–spectral modeling and high fidelity. The core building block, the Quasi-Recursive Attention Unit (QRAU), employs lightweight 2-D convolutions for spatial feature extraction and integrates quasi-recursive pooling with multi-head spectral attention to capture spectral dependencies, effectively suppressing noise. In parallel, an adaptive cross-layer skip connection strategy, incorporating channel-wise concatenation and transition blocks, is introduced to enable efficient multi-level feature fusion within an asymmetric encoder–decoder architecture, balancing spatial detail preservation and spectral fidelity. Experiments on synthetic and real datasets show that QRSAN consistently outperforms existing methods in visual and quantitative evaluations. Specifically, on synthetic data, QRSAN achieves the highest PSNR and SSIM and the lowest SAM across various noise levels, validating its ability to recover both spatial and spectral information; on real datasets, it maintains superior denoising performance and detail preservation. Ablation studies further confirm that QRAU effectively models spatial–spectral dependencies and enhances generalization, while the cross-layer skip connections facilitate robust feature propagation and integration. Overall, QRSAN exhibits both theoretical novelty and practical efficacy. Future work will explore its applications in HSI classification and target detection and other downstream tasks.

## Figures and Tables

**Table 1:** Average results of different methods on the ICVL dataset under various Gaussian noise levels. PSNR and SSIM (↑, higher is better) and SAM (↓, lower is better) are reported. The best values in each row are highlighted in bold.

σ	Index	Noisy	BM4D	NGMeet	LRTF$L_{0}$	QRNN3D	T3SC	MAC-Net	SST	QRSAN
30	PSNR ↑	18.59	37.04	39.42	39.33	39.48	41.13	39.51	42.12	**42.94**
	SSIM ↑	0.1110	0.9287	0.9066	0.9215	0.9498	0.9525	0.9503	0.9718	**0.9739**
	SAM ↓	0.6840	0.2317	0.1201	0.1013	0.0941	0.0966	0.0950	0.0503	**0.0421**
50	PSNR ↑	14.15	34.43	38.81	38.58	38.29	39.47	38.66	40.19	**40.61**
	SSIM ↑	0.0484	0.9159	0.9042	0.9137	0.9309	0.9338	0.9312	0.9566	**0.9572**
	SAM ↓	0.8685	0.3680	0.2475	0.1887	0.0968	0.0991	0.0973	0.0596	**0.0511**
70	PSNR ↑	11.23	32.29	37.44	37.14	36.33	37.46	37.2	38.06	**38.49**
	SSIM ↑	0.0267	0.9014	0.8981	0.9003	0.9008	0.9118	0.9121	0.9372	**0.9383**
	SAM ↓	0.9941	0.5286	0.3329	0.2510	0.1280	0.1097	0.1013	0.0820	**0.0602**
Blind	PSNR ↑	16.54	35.24	38.9	38.76	38.95	39.82	38.83	41.21	**41.73**
	SSIM ↑	0.1039	0.9189	0.9060	0.9171	0.9356	0.9381	0.9377	0.9593	**0.9612**
	SAM ↓	0.7687	0.2617	0.1383	0.1222	0.0997	0.0981	0.0963	0.0562	**0.0493**

**Table 2:** Average results of different methods on the ICVL dataset under five complex noise scenarios. PSNR and SSIM (↑, higher is better) and SAM (↓, lower is better) are reported. The best values in each row are highlighted in bold.

Method	Index	Noisy	BM4D	NGMeet	LRTF$L_{0}$	QRNN3D	T3SC	MAC-Net	SST	QRSAN
Non-iid Gaussian	PSNR ↑	17.79	35.57	33.82	34.06	42.08	41.91	38.36	43.54	**44.07**
	SSIM ↑	0.1617	0.8873	0.8880	0.9054	0.9702	0.9636	0.9573	0.9713	**0.9782**
	SAM ↓	0.7916	0.1183	0.0697	0.0592	0.0493	0.0511	0.0775	0.0344	**0.0334**
Stripe	PSNR ↑	17.75	35.03	33.80	34.13	41.91	41.62	38.41	43.23	**43.95**
	SSIM ↑	0.1596	0.8829	0.9180	0.9275	0.9689	0.9622	0.9551	0.9702	**0.9781**
	SAM ↓	0.7838	0.1416	0.0954	0.1027	0.0508	0.0550	0.0776	0.0362	**0.0349**
Deadline	PSNR ↑	17.68	33.17	32.51	32.93	41.91	39.62	36.41	43.02	**43.89**
	SSIM ↑	0.1622	0.8720	0.8503	0.8778	0.9693	0.9629	0.9538	0.9705	**0.9773**
	SAM ↓	0.7942	0.1673	0.1447	0.1267	0.0510	0.0835	0.1028	0.0365	**0.0343**
Impulse	PSNR ↑	15.11	29.07	28.19	29.25	39.79	36.92	33.61	40.90	**41.65**
	SSIM ↑	0.1228	0.7394	0.8599	0.8619	0.9454	0.9360	0.9261	0.9492	**0.9597**
	SAM ↓	0.8416	0.3496	0.3617	0.3053	0.0814	0.1637	0.2016	0.0618	**0.0594**
Mixture	PSNR ↑	14.24	27.10	26.63	27.41	39.06	34.56	30.39	38.86	**40.89**
	SSIM ↑	0.1022	0.7237	0.8856	0.8881	0.9385	0.9303	0.8965	0.9408	**0.9581**
	SAM ↓	0.8549	0.3628	0.3821	0.3277	0.0840	0.1805	0.2657	0.0619	**0.0607**

**Table 3:** Average results of different methods on the Realistic dataset. PSNR and SSIM (↑, higher is better) and SAM (↓, lower is better) are reported. The best values in each row are highlighted in bold. The * indicates that the improvement of QRSAN over SST is statistically significant according to a paired *t*-test (p<0.05).

Index	Noisy	BM4D	NGMeet	LRTF$L_{0}$	QRNN3D	T3SC	MAC-Net	SST	QRSAN
PSNR ↑	23.26	29.02	28.73	28.85	28.18	28.56	29.24	29.51	**29.94** *
SSIM ↑	0.7609	0.9471	0.9511	0.9522	0.9066	0.9323	0.9488	0.9509	**0.9577** *
SAM ↓	0.3025	0.0539	0.0477	0.0473	0.0976	0.0769	0.0751	0.0463	**0.0440** *

**Table 4:** Ablation study on the contribution of QRAU and its variants on the ICVL dataset under Gaussian noise. PSNR and SSIM (↑, higher is better), SAM (↓, lower is better).

Module	Params (M)	PSNR ↑	SSIM ↑	SAM ↓
RES2D	0.16M	36.26	0.8690	0.0825
QRU2D	0.40M	38.92	0.9283	0.0681
QRU3D	1.18M	40.40	0.9416	0.0609
LSTM	1.63M	40.47	0.9481	0.0575
QRAU	1.19M	40.61	0.9572	0.0511

**Table 5:** Ablation study on the impact of skip connection strategies on the ICVL dataset under Gaussian noise. PSNR and SSIM (↑, higher is better), SAM (↓, lower is better).

Module	Params (M)	PSNR ↑	SSIM ↑	SAM ↓
N-net	0.40M	38.32	0.9127	0.0793
V-net	1.18M	39.19	0.9366	0.0637
C-net	1.21M	39.44	0.9553	0.0555
QRSAN	1.19M	40.61	0.9572	0.0511

## Data Availability

The datasets generated and analysed during the current study are available from the corresponding authors on reasonable request.
